# Combined cone-beam CT imaging and microsurgical dissection of cadaver specimens to study cerebral venous anatomy: a technical note

**DOI:** 10.1007/s00276-023-03195-8

**Published:** 2023-08-05

**Authors:** Markus E. Krogager, Rasmus H. Dahl, Lars Poulsgaard, Kåre Fugleholm, Tom Sehested, Ronni Mikkelsen, Jørgen Tranum-Jensen, Tiit I. Mathiesen, Goetz Benndorf

**Affiliations:** 1 Department of Neurosurgery, University Hospital of Copenhagen, Rigshospitalet, Copenhagen, Denmark; 2 Department of Radiology, University Hospital of Copenhagen, Rigshospitalet, Copenhagen, Denmark; 3 Department of Radiology, Hvidovre Hospital, Copenhagen, Denmark; 4 Department of Neurosurgery, Aarhus University Hospital, Aarhus, Denmark; 5 Department of Neuroradiology, Aarhus University Hospital, Aarhus, Denmark; 6 Department of Biomedicine, Aarhus University, Aarhus, Denmark; 7 Department of Cellular and Molecular Medicine, The Panum Institute, University of Copenhagen, Copenhagen, Denmark; 8 Department of Clinical Medicine, University of Copenhagen, Copenhagen, Denmark; 9 Department of Clinical Neuroscience, Karolinska Institute, Stockholm, Sweden; 10 Department of Radiology, Baylor College of Medicine, Houston, TX USA

**Keywords:** Neuroanatomy, Neuroradiology, Veins, Cadaver, Latex, Barium

## Abstract

**Purpose:**

Cadaver dissections and X-ray based 3D angiography are considered gold standards for studying neurovascular anatomy. We sought to develop a model that utilize the combination of both these techniques to improve current tools for anatomical research, teaching and preoperative surgical planning, particularly addressing the venous system of the brain.

**Materials and methods:**

Seven ethanol-fixed human cadaveric heads and one arm were injected with a latex-barium mixture into the internal jugular veins and the brachial artery. After the ethanol-based fixation, specimens were scanned by high-resolution cone-beam CT and images were post-processed on a 3D-workstation. Subsequent, microsurgical dissections were performed by an experienced neurosurgeon and venous anatomy was compared with relevant 3D venograms.

**Results:**

Latex-barium mixtures resulted in a homogenous cast with filling of the cerebral venous structures down to 150 μm in diameter. The ethanol-based preparation of the cadaveric brains allowed for near-realistic microsurgical maneuverability during dissection. The model improves assessment of the venous system for anatomical education and hands-on surgical training.

**Conclusion:**

To our knowledge we describe the first preparation method which combines near-realistic microsurgical dissection of human heads with high-resolution 3D imaging of the cerebral venous system in the same specimens.

**Supplementary Information:**

The online version contains supplementary material available at 10.1007/s00276-023-03195-8.

## Introduction

Detailed knowledge about neurovascular anatomy is essential in the field of clinical neuroradiology and neurosurgery. Vascular injection and dissection of cadaveric brains are basic methods for studies of microsurgical anatomy [1–5]. Meanwhile, catheter-based digital subtraction angiography (DSA) [6–8] and modern CT and MR angiography provide high-quality images of neurovascular anatomy [9, 10]. Multiple studies have improved the understanding of neurovascular anatomy by combining cadaver dissections with radiography and CT imaging [11–14]. These studies have focused on arterial [10, 14–19] and venous anatomy [10, 11, 15, 20] using iodine, magnetite, or barium-based contrast media for radiographic visualization.

With the advent of endovascular techniques and growing attention to potential complications following venous sacrifice, a detailed understanding of the cerebral venous anatomy has been recognized as essential for interventional neuroradiologists and neurosurgeons [6, 8]. Therefore, we aimed to develop a method that combines 3D imaging using high-resolution cone-beam CT and subsequent microsurgical cadaver dissection for thorough understanding and precise mapping of the cerebral venous anatomy. We further present a novel method for cadaveric preparation that permits extensive filling of the cerebral venous system down to vessels 150 μm in diameter and maintains realistic haptic qualities of the brain during dissections.

## Material and methods

### Ethical considerations and cadaver management

The study was performed on seven human cadaveric heads and one human cadaveric arm from deceased individuals, who bequeathed their bodies to science and education at the Department of Cellular and Molecular Medicine (ICMM), University of Copenhagen. The bodies were handled in accordance with the Danish Health Care Act, #548 §188.

The study was approved by the head of the Body Donation Program at ICMM. Ethical committee permission was not necessary as the individuals had donated their bodies to scientific purposes. Only age and gender of the individuals were known to the investigators.

### Latex-contrast media mixtures

Latex (Ward’s Science, Rochester, NY, USA) was mixed with various concentrations (75%, 50% and 25% v/v of contrast media) of iodine (GE Healthcare, Omnipaque™ 350 mgI/mL) or with barium sulphate (E-Z-EM, Liquid Polibar Plus, 105% w/v, 58% w/w) in order to obtain a suitable radiopaque injectate for radiography and microsurgical dissection. Liquid contrast agents were used throughout this study because these are easier to mix than powder formulations. The mixtures were filled into thin-walled polyethylene plastic tubes of various luminal diameters followed by cone-beam CT scanning. Latex-iodine mixtures were suboptimal due to separation between the latex and iodine solutions. A latex-barium mixture with 25% v/v barium and 75% v/v latex showed no separation, had satisfying solidification properties and was resistant to manipulation during dissection. Accordingly, this latex-barium (75/25%) injectate was chosen for all cadaveric injections. The injectate was initially tested in a cadaveric arm.

### Cadaveric arm model

A 3 mm plastic catheter was inserted into the brachial artery of a fresh-frozen and thawed cadaveric arm and a tourniquet was tightened distal to the transection surface. The latex-barium mixture was injected until a steep rise in resistance was felt—an indication that the vasculature was filled close to the arteriolar level. The arm was stored in 30% ethanol at 4 °C until imaging.

### Cadaveric head model

Cadavers obtained within 48 h postmortem were prepared submerged in tap water in a stainless-steel tub. By bilateral cervical dissection, the internal carotid arteries (ICA) and internal jugular veins (IJV) were exposed, and the vertebral arteries were clamped. The ICAs were catheterized with Foley catheters **(**12 Ch), while the IJVs were left open. A maximum of 200 mL vacuum degassed saline was gently infused through the ICAs to wash out larger clots. Afterwards the brains were fixed by gentle simultaneous injection of both ICAs with 400–600 mL of fixative consisting of 60% ethanol and 10% glycerol (i.e., about 0.5 mL fixative pr. gram brain tissue), resulting in a concentration of about 30% ethanol in the brain tissue sufficient to prevent microbial growth [21] and autolytic processes. Saline wash and fixative infusion was carried out with handheld 100 mL syringes. Forceful saline wash and fixative infusions were avoided to prevent development of cerebral edema. Then, the IJVs were catheterized with Foley catheters (16 Ch) up to the level of the jugular bulb. All steps of dissection, catheterization and injection were carried out under water to prevent air embolies.

In the third to seventh cadaveric heads, a tourniquet was placed around the neck just proximal to the catheter entrances. The necks were then transected below the indwelling catheters and the heads were stored in 30% ethanol for about one week to complete fixation before latex injection.

All seven cadaveric heads were submerged in a large bucket with tap-water and injected through their IJV catheters with a mixture of 75% blue latex and 25% barium (120–160 mL of injectate). The two IJV catheters were injected simultaneously with the blue latex-barium mixture until outflow of the injectate was observed from the internal vertebral venous plexus. The injection was done while observing the spinal cord to avoid excessive intracranial pressure (see online supplement for a more detailed description of the preparation technique).

### Imaging and post-processing

Imaging of the cadaveric specimens was performed with the Artis Q biplane system (Siemens Healthineers, Erlangen, Germany). High-resolution plain radiographs in standard and oblique views and cone-beam CTs (DynaCT) with various field of views (22 and 48 cm) were performed. Cone-beam CT parameters were 20 s acquisition time, 0.4° increment, and 496 total projections. Post-processing was performed by a senior interventional neuroradiologist on a dedicated workstation (Leonardo, Siemens Healthineers) using various kernels to create secondary reconstructions with smaller volumes of interest (VOI). Data were presented as multiplanar reconstructions (MPR), maximum intensity projections (MIP) and with volume rendering technique (VRT). Reading of images was done simultaneously by a neuroradiologist and a neurosurgeon while dissecting the specimens. Images were analyzed via cross-referencing venograms and cadaver dissection via visual cues based on anatomic understanding as illustrated in Fig. 3b, c.

### Microsurgical dissection

After imaging, the heads were stored once again in 30% ethanol until dissection was performed. Microsurgical dissection was performed with a surgical microscope (Zeiss, OPMI S3B surgical microscope, Jena, Germany). Relevant neurosurgical approaches were tested to study and compare surgical venous anatomy with pre-dissection 3D imaging. At least one senior neurosurgeon and neuroradiologist was involved in dissection and reading of images for each specimen.

## Results

### Cadaveric arm

**Fig. 1:**
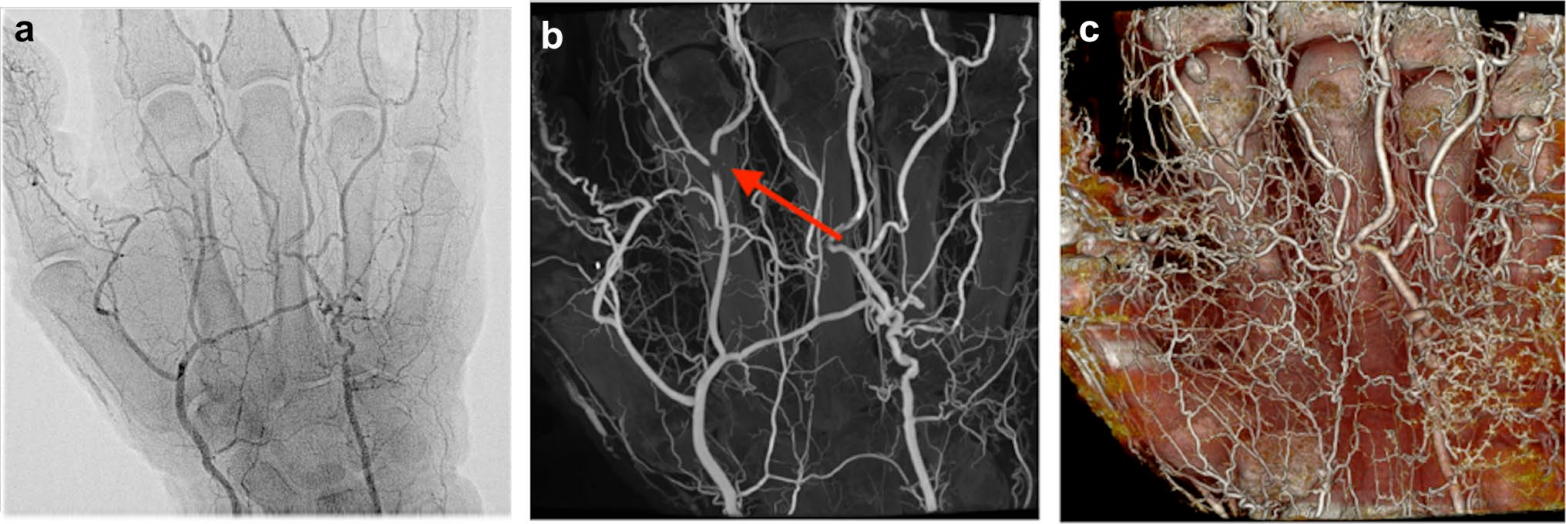
**a** Anterior–posterior projections of a left cadaveric hand injected with a 25% latex-barium mixture into the brachial artery. High-resolution visualization of the superficial palmar arch and digital arteries. **b** Maximum intensity projections (MIP) reconstructed from a cone-beam CT. Arrow pointing at small filling defect. **c** Volume rendering technique (VRT) showing the extensive deep and superficial vascularization of the hand. Note the more detailed visualization of the smallest arteries

Injection of latex-barium mixture into the brachial artery resulted in high-quality visualization of the palm and fingers (Fig. 1a–c). Only small filling defects were noted (Fig. 1b), and the quality of the tissue was suitable for microdissection showing latex filling of intramuscular arterial branches.

### Cadaveric heads

Imaging of the first two cadaver heads, injected without a neck tourniquet, showed irregular and insufficient filling of small cerebral veins and some portions of the sinuses. The lack of distal filling was thought to be caused by pooling of the latex-barium in suboccipital veins and outflow of latex-barium at the transection surface via neck collaterals resulting in sparse intracranial venous filling.

**Fig. 2:**
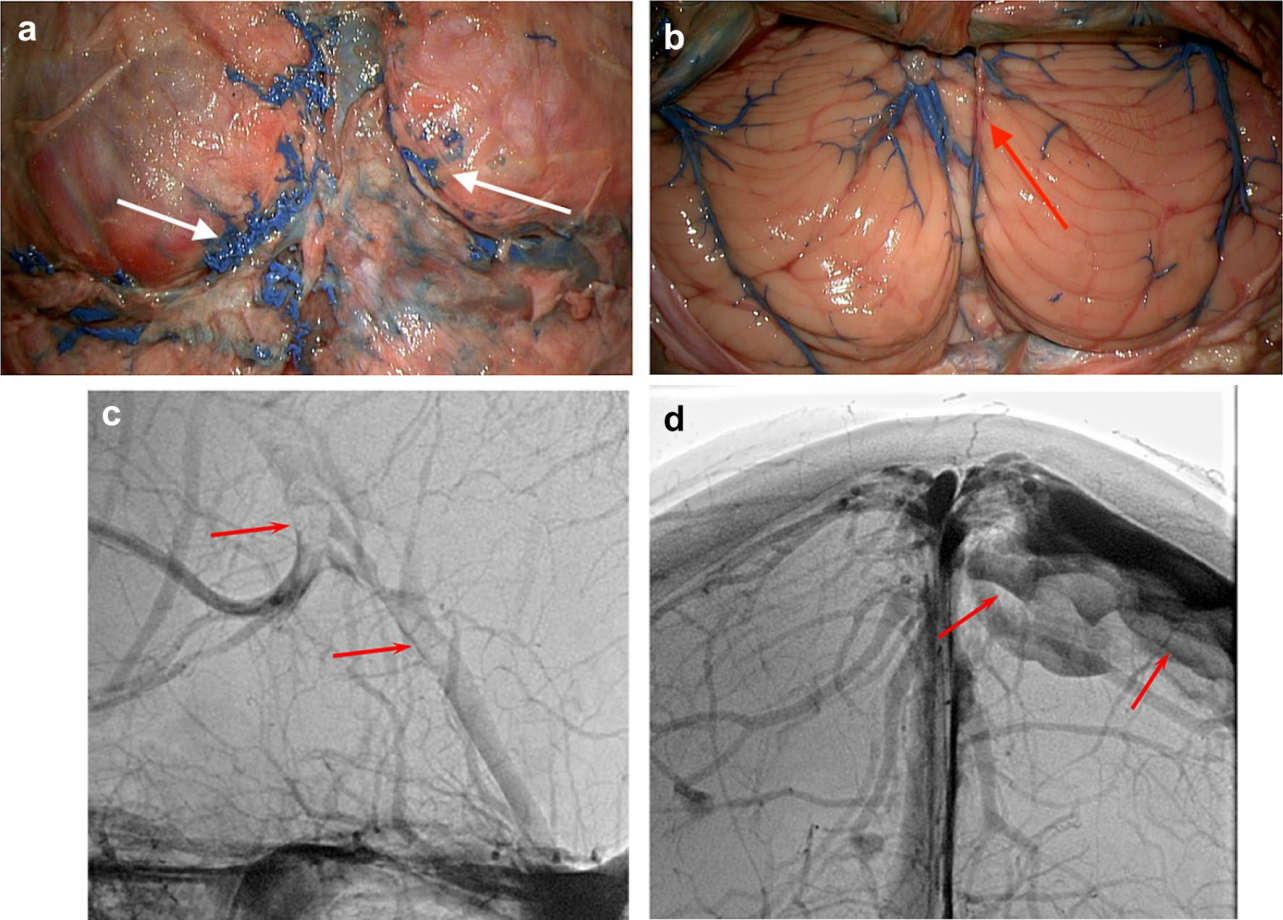
**a** Posterior view of the cerebellum and confluence region during dissection in specimen. Before durotomy, smaller strings of extradural free extravasations of injectate around the confluence, superior sagittal sinus and bilateral transverse sinuses via Paccionean granulations (white arrows). **b** Posterior view of the cerebellar hemispheres after durotomy showing a filling defect (red arrow) in the midline: in the inferior vermian vein on the right side. **c** Radiograph, lateral view shows filling defects in the Vein of Galen as well as the straight sinus (red arrows). **d** Radiograph, AP view shows extravasation (leakage) from the superior sagittal sinus or cortical veins (red arrows) (color figure online)

In addition, filling of deep distal cerebral veins appeared uneven, possibly due to brain edema caused by flushing of the ICAs with saline under too high pressure. Therefore, the volume of saline was reduced, and saline was injected more slowly. Cadaver heads injected after application of a neck tourniquet showed improved cerebral venous filling. Though, elevated pressure during injection resulted in extravasations of the radiopaque injectate in two heads. Extravasations were encountered in relation to intra- and extracranial dural surfaces (Fig. 2a, d).

**Fig. 3:**
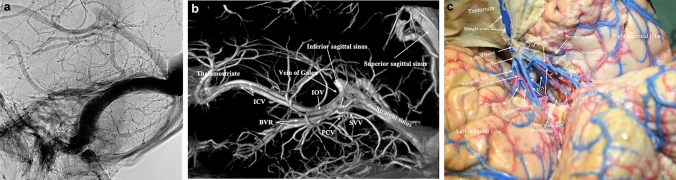
**a** Lateral projection of a cadaveric head injected with a 25% latex-barium mixture into the internal jugular veins. High-resolution visualization of the Galenic system and posterior fossa veins. **b** Volume rendering technique (VRT) after cone-beam CT showing the same anatomy in 3D with significantly more details. **c** Surgical dissection of the same specimen showing the same anatomy in posterior view. Arterial injection was done with red latex and no barium

For each cadaver head, radiographic images of both the superficial and the deep venous systems of the brain showed filling of veins down to 150 $\mathrm{\mu m}$ in diameter (Fig. 3a, b). With the optimized injection technique, the heads had a more homogenous cast allowing better discrimination between vascular structures and brain tissue during dissection. The venous anatomy in the quadrigeminal cistern was chosen as the area of reference among the seven scanned and dissected heads. We managed to identify both larger vessels and sinuses and smaller vessels and tributaries on parallel dissection images and 3D reconstructions as shown in Fig. 3b, c. During dissection, the brain preparation allowed realistic retraction and manipulation during microsurgical instrumentation and was found to be a suitable model to practice neurosurgical approaches. Dissections carried out more than 6 months after cadaver preparation and injection had these qualities preserved.

## Discussion

We have developed a method to conserve cadaveric brains allowing direct cross-validation of 3D imaging and surgical dissection of the cerebral venous system.

In neurosurgery, anatomical variations are increasingly recognized as a potential cause to suboptimal surgical planning, treatment, and complications. The increased risk of unexpected complications is particularly evident when the surgical anatomy involves venous structures [22–27]. In such cases, even experienced neurosurgeons hold remarkably conflicting beliefs on risk and benefit of venous sacrifice for expansion of the surgical window [24, 28–31]. Similarly, expanding knowledge and understanding of cerebral venous anatomy have recently played an increasing role in the endovascular community. Aiming for more precise localization and understanding of arteriovenous shunting lesions as well as to facilitate the access for transvenous embolization techniques has become more important in recent years. The cerebral venous system is highly variable and requires a more individualized approach when examined and defined. In vivo visualization of venous anatomy by intra-arterial DSA, the current gold standard, suffers from flow- and territory dependent filling which is often incomplete and not sufficiently reliable.

No other studies in the literature have so far described a method for comparative visualization and analysis of high-resolution 3D venograms and near-realistic microsurgical anatomy.

### Chemical composition of the brain fixative

Alvernia et al. [15] introduced a method using colored latex media for vascular injection of cadaver heads prior to neurosurgical training. However, the cadaver heads in the study of Alvernia [15] were not perfused with any fixative. Unfixed brain tissue only retains its haptic qualities in a limited time frame [16], and is therefore unsuitable for combined imaging and repeated investigation of anatomy in multiple dissections.

In three recent studies on cadaver head preparation with vascular injections, a fixative containing formaldehyde was used to retain the structural qualities of the brain tissue [10, 11, 17]. However, formaldehyde hardens the tissue [16] resulting in a loss of maneuverability and impairs the natural haptic qualities of the brain tissue. Therefore, we chose a formulation containing only ethanol and glycerol, similar to the one used by Zhao et al. [14]. High concentrations of ethanol are also known to have a fibrinolytic effect on blood clots [32].

### Mixing the colored injectate with contrast media

Burchianti et al. [11] developed a method of cadaver injection for comparison of radiographic vascular anatomy with surgical skull base anatomy. Their injectate contained latex and iodinated contrast, and both arteries and veins were injected. Standard CT scans showed inconsistent filling of the radiopaque injectate in the venous structures. Viewing the dissection images, it appeared that the authors had issues with latex hardening, mixture separation and diffusion of iodine through the vascular wall. During our initial tests, we experienced similar problems.

Zhao et al. [14] presented an injection method that combined colored silicone and iodinated contrast media (Renografin 60) to visualize arterial anatomy to improve surgical planning and found that at least 25% iodinated contrast agent was necessary for sufficient radiopacity. Their injectate did also show signs of separation. We found that our latex-based technique offers lower cost and easier handling during injections compared to silicone solutions [15].

Turkoglu et al. [10] tested two mixtures containing silicone rubber mixed with either powdered barium or tantalum in 20 cadavers. The authors found significant angiographic irregularities with the mixtures containing powder. Though, they had good results in a third mixture, where gelatin was mixed with liquid barium. In our opinion, a liquid barium-gelatin mixture is less suitable for microsurgical dissection due to the lower elasticity and strength of the hardened injectate. Sedlmayr et al. [12] tested injections in goose heads using a mixture of 25% barium sulphate and 75% latex with satisfying results, which we were able to reproduce in the present study.

### Evolving radiographic technology calls for new methods of radiological investigation

While most studies [12, 13, 20] used radiographs for studying injectates of colored agents combined with contrast agents, Burchianti et al. [11] used conventional CT-based imaging. Zhao et al. [14] was not able to clearly separate vessels from bone on MIP images due to the overlap of the density of bone and injected vessels. Turkoglu et al. [10] previously used cone-beam CT imaging of cerebral arteries. The latex-barium mixture and high-resolution cone-beam CT for visualization of cranial venous anatomy has, to the best of our knowledge, not previously been described. Moreover, we optimized reported injections techniques by maintaining strict submersion of the bodies for prevention of air emboli and application of a tourniquet to prevent loss of injectate to extracranial veins resulting in impaired intracranial venous filling.

### Limitations and future perspectives

Extravasations of the radiopaque injectate impaired 3D visualization in certain locations (Fig. 2d). This can possibly be avoided by improving positioning of the catheter before injection to confirm that the catheter is not blocked by a blood clot risking sudden excessive pressure on the syringes.

Intraluminal filling defects may be caused by compression of thin-walled vessels from cerebral edema, residual fixative, saline or blood clots (Fig. 2b, c).

A selection of venous structures of all sizes was only identified and cross-validated between 3D reconstructions and dissection around the quadrigeminal cistern. This was done to systematically assess the quality of the technique and complications as those mentioned above. The posterior fossa, pineal region and the confluence area is representable for the variance among venous structures.

Combined 3D reconstructions of both the venous system and the arterial system, as reported by Burchianti et al. [11], would be possible and could be implemented when needed. Initial testing of our injection technique on an arm showed detailed vessel filling and vascular presentation during dissection meaning that this technique can be used both venously and arterially in other vascular territories.

Continuous refinement of methods to obtain detailed 3D venograms combined with near-realistic microsurgical approaches may help to improve the understanding of the cerebral venous anatomy and its variations. High-resolution venous 3D imaging may potentially be used to predict consequences of impaired venous drainage, and thus improve surgical safety [33].

## Conclusions

We present a novel method that combines high-resolution 3D radiographic visualization and microsurgical dissection of the cerebral venous anatomy in human cadaveric heads using a radiopaque latex-barium injectate. The method was easy to reproduce and allowed deep penetration into small vessels. It allowed for direct comparison of high-resolution 3D imaging with microsurgical dissection and may provide a promising new tool to improve and expand the understanding of cerebral and skull base venous anatomy in anatomic education, surgical planning and training, and neurovascular research.

### Supplementary Information

Below is the link to the electronic supplementary material.

Supplementary file1 (DOCX 25 kb)

## Data Availability

A detailed description of the injection technique presented in this manuscript can be found in the supplementary material.

## Abbreviations

DSA: Digital subtraction angiography
ICA: Internal carotid artery
IJV: Internal jugular vein
VOI: Volumes of interest
MPR: Multiplanar reconstructions
MIP: Maximum intensity projections
VRT: Volume rendering techniques

## Declarations

### Previous presentations

The content of this project including the figures was presented as a poster at the eEANS2021 (available at https://www.researchgate.net/publication/364585342_Latex-Barium_injection_in_cadaver_heads_for_combined_macroscopic_and_3D-radiographic_anatomical_studies_A_Technical_Note).
