# Supplementary material for: Combined cone-beam CT imaging and microsurgical dissection of cadaver specimens to study cerebral venous anatomy: a technical note
Source: Surg Radiol Anat. 2023 Aug 5;45(9):1177–84. doi: 10.1007/s00276-023-03195-8 (PMC10514096; doi:10.1007/s00276-023-03195-8)
Supplement: Supplementary file 1 — Supplementary file1 (DOCX 25 kb) [file 276_2023_3195_MOESM1_ESM.docx]

**Supplement**

A step-by-step guide to latex-barium injections in cadaver heads

**Step 1: Handling of the corpse**

The non-frozen fresh cadaver, obtained within 48 hours *postmortem* is lowered in its entirety into a tub filled with tepid tap water until the neck is fully submerged to prevent introduction of air embolies during neck dissection and catheter insertion. The corpse may need to be held submerged by weights to allow stable submersion.

**Step 2: Neck dissections and carotid catheterization/cannulation**

While still submerged, dissection is carried out to expose the internal jugular veins (IJV), the common carotid arteries (CCA) and the carotid bifurcations. The vertebral arteries are exposed and preferably clamped just caudal to the transverse process of C6. Clamping of the vertebral arteries improves the fixation of cerebellum.

The distal end of two Foley catheters (12 Ch) are cut off (including the balloon) and the catheters are filled with water, submerged, and ensured that they are free of air bubbles. The CCAs are cut open as caudal as possible. The catheters are inserted and advanced until situated in the internal carotid arteries. A ligature is placed tightly around each vessel and the indwelling catheters. The IJVs are cut open.

**Step 3: Arterial saline flushing**

100 mL syringes with vacuum-degassed saline are connected to carotid catheters and saline is gently infused simultaneously while observing the outflow from the IJVs exit of clots. It is essential that the connection of the syringes to the catheters is performed during submersion to prevent introduction of air during assembly.

**Step 4: Tourniquets placement and venous saline flushing**

The IJVs are then catheterized like the arteries using water-filled Foley catheters (16 Ch) inserted up to level with the jugular bulbs and ligated. A neck tourniquet is placed cranially to the vascular entrance of the catheters and caudally to the catheter's intravascular cranial ends. The tourniquet is tightened to minimize filling of the cervical and suboccipital venous plexus during fixation and the later latex-barium injection. With arterial catheters closed, the venous flushing procedure is now carried out through the IJV by gentle infusion of saline, first on the right and then on the left side with the contralateral catheter open. The cerebral vasculature should now be cleared of blood clots. It is essential not to force the injection of saline and the total infused volume should not exceed 200 mL saline in total to avoid cerebral edema.

**Step 5: Ethanol fixation**

Fixation is done with infusion of a water-based 60% ethanol and 10% glycerol solution infused through the cannulated arteries. All four catheters are left open and kept submerged in the tub before the start of fixation. A minimum of 400 mL of fixative is injected until fixative flows from both IJV catheters. After fixation, all the catheters are clamped tightly.

**Step 6: Neck transection**

The tub is emptied of water and the corpse is placed on a trolley. The head is detached from the torso below the four inserted catheters and stored for 8-10 days at 4^o^C in 30% ethanol to complete the fixation and give time for a possible fibrinolytic action of ethanol.

Neck transection is performed before the latex-barium injection to allow evaluation of the intracranial pressure during injection.

**Step 7: Venous latex-barium injection**

After minimum a week to complete fixation, the catheterized head and neck is submerged once again in tap water, upside-down, with the transected surface just below the surface. We used a bucket and a silicone ring to stabilize the head in this position.

Prepare 2-3 x 60 mL syringes with a 25% barium-sulphate and 75% blue latex mixture (v/v). A three way stop cock can be used for mixing the injectate. It is important to obtain a homogenous injectate without air bubbles. The clamps are removed on all four catheters with the specimen submerged. Then, the two IJV catheters are injected simultaneously with the blue latex-barium mixture until outflow of the injectate is observed from the internal vertebral venous plexus. The injection is done gently while observing that the spinal cord position at the transected surface, is stable in the vertebral canal to avoid excessive intracranial pressure and risk of incarceration. Usually, 120-160 mL of injectate is required.

**Step 8: Arterial latex injection (optional step)**

After at least 2 hours of latex hardening, a similar injection procedure may be carried out through the two carotid arteries using red latex without barium. The arteries are injected one at a time until contralateral outflow is observed. Usually, 60-80 mL of injectate is required on arterial side. This step allows identification of arteries during subsequent microsurgical dissection.

**Step 9: Storage until further examination**

The injected head is stored in 30% ethanol-solution for latex hardening and conservation until radiologic examination or dissection is performed. The neck catheters should remain closed until latex hardening (or up to 24 hours).
